# Reagents and models for detecting endogenous GLP1R and GIPR

**DOI:** 10.1016/j.ebiom.2021.103739

**Published:** 2021-12-12

**Authors:** Julia Ast, Johannes Broichhagen, David J. Hodson

**Affiliations:** a Institute of Metabolism and Systems Research (IMSR), Centre of Membrane Proteins and Receptors (COMPARE), University of Birmingham, Birmingham, UK; b Centre for Endocrinology, Diabetes and Metabolism, Birmingham Health Partners, Birmingham, UK; c Leibniz-Forschungsinstitut für Molekulare Pharmakologie, Berlin, Germany

**Keywords:** GLP1R, GIPR, GLP1, GIP, incretin-mimetics, diabetes, pancreas, brain, GLP-1R, GLP-1

## Abstract

Glucagon-like peptide-1 receptor (GLP1R) agonists target the GLP1R, whereas dual GLP1R/ gastric inhibitory polypeptide receptor (GIPR) agonists target both the GLP1R and GIPR. Despite the importance of these drug classes for the treatment of diabetes and obesity, still very little is known about the localization of GLP1R and GIPR themselves. Complicating matters is the low abundance of GLP1R and GIPR mRNA/protein, as well as a lack of specific and validated reagents for their detection. Without knowing where GLP1R and GIPR are located, it is difficult to propose mechanisms of action in the various target organs, and whether this is indirect or direct. In the current review, we will explain the steps needed to properly validate reagents for endogenous GLP1R/GIPR detection, describe the available approaches to visualize GLP1R/GIPR, and provide an update on the state-of-art. The overall aim is to provide a reference resource for researchers interested in GLP1R and GIPR signaling.

## 1 Introduction

The glucagon-like peptide-1 receptor (GLP1R) and gastric inhibitory polypeptide receptor (GIPR) are G protein-coupled receptors belonging to the secretin receptor super-family, also known as class B [1, 2]. Classically, GLP1R and GIPR are considered to mediate the ‘incretin effect’, whereby glucose leads to a larger rise in insulin secretion when administered orally versus intravenously due to release of glucagon-like peptide-1 (GLP1) and gastric inhibitory peptide (GIP) from gut enteroendocrine cells 3, 4, 5. These clinical observations, together with efforts to clone the receptors [6, 7] and identify their stabilized ligands, led to development of the GLP1R agonist class of drugs (or incretin-mimetics) [8]. GLP1R agonists are now a mainstay of type 2 diabetes (T2D) therapy, with the latest drug generation inducing 20-30% weight loss in obese patients [9]. Moreover, placebo-controlled trials have shown potential for GLP1R agonists in the treatment of NAFLD/NASH [10, 11], as well as (possibly) Parkinson's Disease [12], pointing to wider metabolic and neurological actions above and beyond effects on insulin release. Discovery science studies have now shown wide-ranging actions of GLP1R, including on immune cells, neurons, cartilage, lung, adipose tissue and stomach, suggesting future therapeutic targets (reviewed in [13, 14]).

By contrast to GLP1R, the related GIPR has gained much less attention. Part of the reason for this is that infusion of GIP has no effect on insulin secretion in T2D patients, is unable to influence food intake, may be obesogenic, and GIPR is downregulated during T2D/obesity (reviewed in [15]). However, recent studies have shown that GIPR-GLP1R co-agonists or GIPR-GLP1R unimolecular agonists are unexpectedly more effective anti-hyperglycemic and anti-obesity agents compared to GLP1R agonist control [16, 17]. Further complicating the picture, co-administration of a GLP1R agonist and GIPR antagonist, either alone or as a conjugate, demonstrated equally impressive reduction in weight loss in mice and non-human primates [18, 19]. How such synergistic effects occur are poorly characterized, but might include higher potency at the GLP1R, alterations to GLP1R/GIPR internalization or restoration of GIPR signaling following GLP1R-mediated metabolic normalization [15, 20].

Despite the apparently pleiotropic effects of GLP1R agonists, and the impressive performance of GIPR-GLP1R co-agonists in clinical trials, a major problem continues to stalk the field: lack of reliable and validated reagents for detecting GLP1R and GIPR. Despite evidence to the contrary 21, [22], 23, a cursory glance of the literature will reveal that hepatocytes express GLP1R, and that GLP1R/GIPR in their non-stimulated state are predominantly in the cell cytoplasm. Why is this relevant when efficacious GLP1R agonists and dual agonists already exist? Firstly, if we are unable to accurately and specifically localize GLP1R/GIPR, then it is impossible to infer cellular substrates for their effects, preventing discovery of new mechanisms. Secondly, following up spurious mechanisms wastes precious research time and resources. Thirdly, GLP1 can be derived from the gut and brain, yet how the different pools access different body sites remains incompletely understood. Fourthly, how GIP agonists/antagonists/co-agonists access the different brain regions is poorly defined. The aim of the current review is to therefore discuss: 1) the pitfalls of detecting GLP1R/GIPR and how to properly validate reagent specificity; 2) methods to detect GLP1R/GIPR, including antibodies, ligands, reporter mice, with advantages and disadvantages therein; and 3) latest innovations in GLP1R/GIPR detection, including enzyme self-labels, super-resolution imaging and electron microscopy (EM). Ultimately, we hope that the review article will provide a useful update for anyone intending to detect and visualize GLP1R/GIPR in their cell/tissue system of choice.

## 2 GLP1R/GIPR detection pitfalls

In the pancreatic islets—one of the best validated models for GLP1R/GIPR signaling—*GLP1R*/*GIPR* transcript abundance in sorted beta cell fractions is ∼1000-fold lower than *INS* and ∼5-fold lower than *PDX1*, a beta cell-specific transcription factor [24]. In keeping with other membrane receptors (e.g. *Sstr1-5, Ghsr1a, Avpr1b*) expression of *Glp1r*/*Gipr* is relatively low [25, 26], meaning that interpretation of scRNA-seq datasets should proceed with caution: the high drop-out rate of these transcripts gives rise to heterogeneous expression, which is not borne out at the protein level when enriched fractions are carefully examined [27]. Such low expression levels are perhaps to be expected given the highly-amplified nature of GPCR signaling.

Transcriptomic analyses are even more problematic in tissues comprised of diverse, overlapping and fragile cell populations (e.g. brain) where purification is difficult and alternatives such as scNuc-seq or RNAScope are needed [28]. Thus, given the low abundance of *GLP1R*/*Glp1r* and *GIPR*/*Gipr* transcripts, gold-standard detection of GLP1R/GIPR protein expression is likely to be challenging. It stands to reason therefore that controls are paramount when detecting GLP1R/GIPR protein. However, what should these controls look like?

Positive controls should include the pancreas—specifically the pancreatic islets—where GLP1R is localized predominantly to beta cells, and GIPR to alpha and beta cells [27, 29, 30, 31, 32]. Negative controls will depend on species, but when using a reagent for the first time, or attempting to detect GLP1R/GIPR in a new cell type/tissue, knockout tissue should be used to confirm lack of expression. Thoroughly validated *Glpr^−/−^* and *Gipr^−/−^* knockout (KO) mice have been reported and are available from donating investigators or repositories subject to material transfer agreement [29, 33, 34, 35]. *Glp1r*-flox'd and *Gipr*-flox'd mice also exist [36, 37], affording conditional and tissue-specific deletion of *Glp1r*/*Gipr*. While studies in cell lines heterologously-expressing GLP1R/GIPR can be useful, we note that staining/labeling can be cell- and tissue-dependent, and receptor expression levels tend to be much higher in stable/transient transfection systems. A CRISPR-deleted rat INS1 832/3 GLP1R^*−/−*^ beta cell line has been described [38], and could serve as a useful control, since native INS1 832/3 cells endogenously express the receptor albeit at relatively low levels versus primary beta cells [22].

Reagent validation in human tissue is more challenging given the lack of specific GLP1R/GIPR KO tissue. In general, reagents should be tested in human-derived cell lines transfected with and without (human) GLP1R/GIPR, or graded expression of GLP1R/GIPR (i.e. low and high) [21, 39, 40]. Going forward, availability of EndoC-βH human beta cell lines [41] will enable CRISPR deletion or stable lentiviral shRNA knockdown of GLP1R/GIPR, although we note that EndoC-βH1 cells express some key human beta cell genes at low levels, including *DLK1, RGS16, IAPP* and *HDAC9* [42]. While EndoC-βH1 cells mount insulin secretory responses to GLP1R agonist [42][43], and *GLP1R* mRNA can be knocked down [42], we (unpublished data) and others [42] were unable to detect GLP1R protein using antibodies or probes. However, EndoC-βH3 have increased potential for clonal expansion [44], which should allow GLP1R-null lines to be produced in the future. While GLP1R/GIPR knockdown is more difficult in primary human tissue, recent reports have shown that re-aggregated human islets are amenable to CRISPR-deletion [45], which could allow confirmation of reagent specificity in *GLP1R*/*GIPR^−/−^* islets.

In addition to the above, there are a few general guidelines/caveats which should be acknowledged when attempting to detect GLP1R/GIPR: 1) protein abundance in primary tissue is likely to be too low to detect using western blot without a prior immunoprecipitation step; 2) GLP1R/GIPR are 7 transmembrane proteins present at the cell surface in their unstimulated state, so immunostaining/labeling should be observed primarily in this location (with the caveat that constitutive internalization is theoretically possible); and 3) do not rely on specificity of commercial reagents unless they have been thoroughly tested in KO tissue or heterologous cell systems.

Table 1 lists reagents/tools that have been validated using antibody co-localization, known tissue localization/reporters, RNA confirmation, pharmacology or *Glp1r^−/−^* and *Gipr*^*−/−*^ tissue/cells.

**Table 1:** Validated reagents for detection of GLP1R and GIPR in cells and tissue. GLP1R/GIPR antisera, fluorescent agonists/antagonists and mouse models validated according to known cell localizations, enzyme self-labels, pharmacology, *Glp1r/Gipr* expression in enriched fractions, *Glp1r^−^^/^^−^* tissue, *Glp1r^−^^/^^−^* cells or GLP1R/GIPR-transfected cells.

Reagent	Name	Source	Reported cross-reactivity	Validation
GLP1R antibody [39]	Mab 3F52	Iowa DSHB	Human, primate	GL1PR_BHK cells (and wild-type cells)
GLP1R antibody [50]	Mab 7F38	Iowa DSHB	Human, Mouse, Rabbit, Rat	*Glp1r^−/−^* mice
GLP1R antagonistic antibody [27, 48]	Glp1R0017, GLP1R-APC	University of Cambridge, Duke University	Mouse	*Glp1r^−/−^* mice, pharmacology, antibody co-localization
GLP1R antibody [53]	ab218532	Abcam	Mouse, Rat	*Glp1r^−/−^* mice
GLP1R antibody*[18]	MAB2814	RnD Systems	Human	SNAP_hGLP1R-U2OS (and wild-type cells)
GLP1R agonist [57, 58, 60, 61]	E4_K12_-Fl, E4_×12_-VT750, EP12-TR, EP12-BTMR, EP12-BTMR-X, EP40-BF, EP40-TR	Harvard University	Mouse, human	GLP1R_HEK293 cells, MIN6, insulin reporter/staining, pharmacology
GLP1R agonist [59]	E4_x12_-Cy7	Memorial Sloan Kettering Cancer Center	Mouse, human	GLP1R_HEK293, insulin reporter, pharmacology
GLP1R agonist [97]	Ex4-Cy3, Ex4-Cy5	Novo Nordisk	Mouse	*Glp1r^−/−^* mice
GLP1R agonist [65, 66]	Liraglutide^594$^, Liraglutide^750$^, Semaglutide^VT750$^	Novo Nordisk	Mouse, rat	*Glp1r^−/−^* mice
GLP1R agonist 62, 63, 64	Ex4-FITC, Ex4-TMR, lixisenatide‐FITC	Imperial College London	Human	SNAP_GLPR-HEK293, INS-1 832/3 SNAP_GLP1R, pharmacology
GLP1R antagonist [66]	exendin(9-39)^594$^	Novo Nordisk	Mouse, rat	*Glp1r^−/−^* mice
GLP1R antagonist [22, 29]	LUXendin492, LUXendin551, LUXendin555, LUXendin615, LUXendin645, LUXendin651, LUXendin762	University of Birmingham	Mouse/hESC	*Glp1r^−/−^* mice, INS1 832/3 GLP1R^*−/−*^ cells, GLP1R antibody co-localization, pharmacology
GLP1R antagonist [67, 68]	exendin(9-39)-FITC	Imperial College London	Human	SNAP_GLPR-HEK293, pharmacology
Reporter mouse [30]	*Glp1rCre* (transgenic)	University of Cambridge	n/a	*Glp1r* expression, FACS + QPCR
Reporter mouse [55]	*Glp1rCre* (knock-in)	Harvard University	n/a	GLP1R antibody (GLP1R-APC) co-localization [27]
Reporter mouse [31]	*Glp1rCre* (knock-in)	University of Copenhagen	n/a	GLP1R antibody (Mab 7F38) and in situ hybridization
Reporter mouse [32]	*Gipr-Cre* (knock-in)	University of Cambridge	n/a	*Gipr* expression (RNAScope)
GIPR antagonistic antibody*[19]	muGIPR	Amgen	mouse	Pharmacology (not tested for detection)
GIPR antibody*[18]	hGIPR-Ab	Amgen	human	Pharmacology (not tested for detection)
GIPR antibody*[18]	MAB8210	RnD systems	human	SNAP_hGIPR-U2OS (and 0wild-type cells)

⁎ independent verification needed. ^$^Chemical characterization not reported, structures undisclosed.

Table 2 lists the tissues in which the various antibodies/probes have been validated.

**Table 2:** Tissues in which each antibody and probe have been validated. Only reagents shown to stain/label primary tissues are included.

Reagent	Name	Tissues stained or labeled
GLP1R antibody [39]	Mab 3F52	Pancreas, kidney, lung, heart, GI tract
GLP1R antibody [50]	Mab 7F38	Pancreas, kidney, lung, brain (non-fluorescent)
GLP1R antagonistic antibody [27, 48]	Glp1R0017, GLP1R-APC	Pancreas
GLP1R antibody [53]	ab218532	Pancreas, kidney, brain
GLP1R agonist [97]	Ex4-Cy3, Ex4-Cy5	Pancreas
GLP1R agonist [65, 66]	Liraglutide^594^, Liraglutide^750^, Semaglutide^VT750^	Pancreas, brain
GLP1R antagonist [22, 29, 66]	LUXendin492, LUXendin551, LUXendin555, LUXendin615, LUXendin645, LUXendin651, LUXendin762, exendin(9-39)^594^	Pancreas, brain

**Current validated methods to detect GLP1R/GIPR**

## 3 GLP1R/GIPR mRNA quantification and hybridization

Due to drop-out rate of lowly expressed genes when using single cell transcriptomics, alternative approaches should also be used to confirm absence or presence of *GLP1R* and *GIPR* at the transcript/mRNA level. One possibility is to use conventional PCR analyses on purified cell fractions, with primers spanning the *GLP1R*/*Glp1r and GIPR/Gipr* open reading frames (ORFs), and positive controls such as Brunner's gland (GLP1R) or islets (GLP1R/GIPR) [21, 36]. Another possibility is to use bulk RNA-seq on sorted cell fractions, which has shown the utility to identify GPCRs expressed in specific islet cell populations (e.g. ghrelin receptor expressed in delta cells) [25, 26]. These approaches are less amenable to the brain, since neurons are difficult to dissociate and purify. snRNA-seq has proved more fruitful in this tissue, with a number of labs showing *Glp1r* and *Gipr* expression in various neuronal (sub)populations [46, 47]. However, results should ideally be confirmed using in situ hybridization [32], since neurons may be falsely assigned as *Glp1r*-/*Gipr*- given the high transcript drop-out rate. In particular, RNAscope or single molecule FISH have the added advantage of providing spatial information, can be multiplexed for assessment of cell state, and allow single molecule quantification. While measurement of *Glp1r* and *Gipr* at the mRNA level usually maps onto protein expression, it is worth noting that there can be discordance between the two levels, with a study showing that isolated pancreatic delta cells express *Glp1r* mRNA but undetectable protein [27].

## 4 GLP1R/GIPR antibodies

GLP1R: Dozens of GLP1R antibodies are commercially available. To date, only four antibodies have been shown to be specific for detection of GLP1R in mouse, rat, nonhuman primate and human tissue (to the best of our knowledge). Glp1R0017 is a monoclonal antagonistic antibody derived from naïve phage display as a single-chain variable fragment, followed by human IgG1 conversion [48]. Glp1R0017 was found to be a specific GLP1R antagonist using cAMP assays in CHO cells expressing mouse, human, rat, cynomolgus monkey and dog GLP1R [48]. Notably, Glp1R0017 staining co-localized with a GLP1RCre;R26-tdRFP reporter, and was completely absent in *Glp1r^−/−^* tissue [48]. Further confirming the specificity of Glp1R0017, an APC-conjugated version (GLP1R-APC) was shown to enrich islet cells according to their *Glp1r* expression and was unable to label beta cells conditionally deleted for *Glp1r* [27]. Mab 7F38 is another monoclonal antibody, first reported in 2015, and produced by immunizing *Glp1r*^−/−^ mice with mGLP1R_BHK cells, before hybridoma production of antibodies. Specificity of the antibody was subsequently validated using renal vasculature, brain and islets derived from *Glp1r*^−/−^ mice [29, 49, 50, 51]. We note that, while this antibody works for fluorescent immunostaining in islets, there are anecdotal reports that it is less effective in the adult mouse brain, limiting co-localization with neural/glial markers. However, Mab 7F38 has been shown to be effective in mouse and rat brain using non-fluorescent staining [50, 52]. A similar antibody, Mab 3F52, was produced against the human GLP1R extracellular domain and validated in BHK cells with low and high human GLP1R levels [39], subsequently confirmed by a second independent group [21]. Mab 3F52 has been shown to stain primate and human islets, kidney, sinoatrial node, GI tract and thyroid [21, 39]. One of the newest antibodies, Abcam ab218532, shows clear membrane-localized labeling using fluorescent and non-fluorescent immunohistochemistry of pancreatic islets, and was shown to stain wild-type but not *Glp1r^−/−^* tissue [53]. A final antibody, Novus 19400002 was shown to detect human GLP1R in hGLP1R_BHK cells using western blot, but was unable to detect GLP1R in Brunner's gland or pancreas, questioning its sensitivity [21]. Mab 7F38 and Mab 3F52 are freely available on a non-profit basis from Iowa Developmental Studies Hybridoma Bank. A range of other commercial antibodies, widely used in the literature, and some still available to purchase, have been dismissed as non-specific [40].

GIPR: Compared to GLP1R, antibody detection of GIPR is even more challenging. In general, GIPR is expressed at lower levels than GLP1R. Analysis of a published dataset reveals that enriched human beta cell fractions express ∼ 2-fold lower *GIPR* versus *GLP1R* (30.99 ± 20.35 TPM versus 64.57 ± 19.16 for *GIPR* and *GLP1R*, respectively; mean ± SD) [24]. Moreover, GIPR has not been the same focus of industry/academic efforts to produce antibodies, since GIPR agonism/antagonism has only just emerged as a viable option for diabetes/obesity therapy. Nonetheless, the specificity of a number of commercially-available reagents has already been questioned: three antibodies were found to stain HEK cells transfected with empty vector, as well as FLAG-GIPR, despite an anti-FLAG antibody only recognizing the latter [54]. An antagonistic antibody was recently described, termed muGIPR, produced by immunizing mice with plasmid encoding full length mouse GIPR before hybridoma generation. muGIPR antagonized GIPR-mediated cAMP generation, as well as prevented responses to DA-GIP challenge in vivo [19]. The same authors subsequently produced hGIPR-Ab, which displays similar specificity to muGIPR [18]. However, neither of these antibodies was used in the cell or tissue setting to detect GIPR and for the moment remain as tools to modulate GIPR signaling. The same authors did however use two new commercial antibodies against human GLP1R (RnD Systems MAB2814) and human GIPR (RnD Systems MAB8210), showing no detectable staining in non-transfected U2OS cells, but staining in SNAP_hGLP1R-U2OS and SNAP_hGIPR-U2OS, with signals overlapping with SNAP label, thus demonstrating specificity [18]. It will however be important to repeat experiments using U2OS with lower levels of hGLP1R and hGIPR, as well as using human tissue in which known cell-type distributions can be assessed. Going forwards, more GIPR antibodies are likely to become available due to the renewed clinical/discovery science interest associated with development of GLP1R/GIPR co-agonists. Lessons should be learnt from efforts to validate GLP1R antibodies and the same stringent standards applied.

## 5 Reporter animals

GLP1R: The first *Glp1rCre* mice were produced by inserting an iCre flanked by 5’ and 3’ *Glp1r* gene sequences into a murine-based bacterial artificial chromosome (BAC), followed by pronuclear injection of BAC-DNA and random integration into the genome. These mice express Cre-recombinase under the *Glp1r* promoter [30] (MGI ID: 5755096). Two knock-in *Glp1rCre* models exist with IRES-Cre knocked-in downstream of the *Glp1r* gene (i.e. after the stop codon) [31, 55] (JAX stock no. 029283). Following breeding with animals possessing LoxP-flanked reporter alleles (e.g. tdTomato, tdRFP, YFP), Cre-mediated recombination leads to reporter expression only in GLP1R+ cells or their progeny [30, 55]. Since reporter allele expression is usually driven from the Rosa26 locus with a powerful CAG promoter, approaches using *Glp1rCre* animals circumvent the low expression levels of *Glp1r*. Thus, even cell populations with low levels of *Glp1r* expression are likely to be marked with high fidelity, making *Glp1rCre* a useful tool for understanding the localization of GLP1R+ cells/neurons. Moreover, *Glp1rCre* allows GLP1R+ cells to be lineage or fate-mapped, opening up questions such as: do GLP1R+ cells remain GLP1R+ or can they de-differentiate or trans-differentiate to other lineages?

Inherent problems with reporter approaches are: 1) readout of *Glp1r* promoter activity rather than the protein itself; 2) inability to know whether the reporter-positive cell is in fact GLP1R+, or is the progeny of a GLP1R+ cell that has adopted another fate; and 3) lack of information regarding endogenous GLP1R expression levels, as well as orthosteric binding capacity. It is worth noting however that studies have shown excellent overlap between *Glp1rCre*;reporter animals and antibody/probe labeling, meaning that GLP1R+ cell populations are unlikely to be highly plastic during development [27, 30, 31]. While the transgenic model failed to label acinar cells in the pancreas [30], in contrast to one of the knock-in models [31], this might reflect the very low reported GLP1R/*Glp1r* expression levels in exocrine tissue [39, 56]. Nonetheless, *Glp1rCre*;reporter animals are arguably the highest fidelity model available for detection of GLP1R, allowing resolution of small cell subpopulations which would be difficult to identify with antibody approaches (e.g. pancreatic duct cells).

**Fig. 1:**
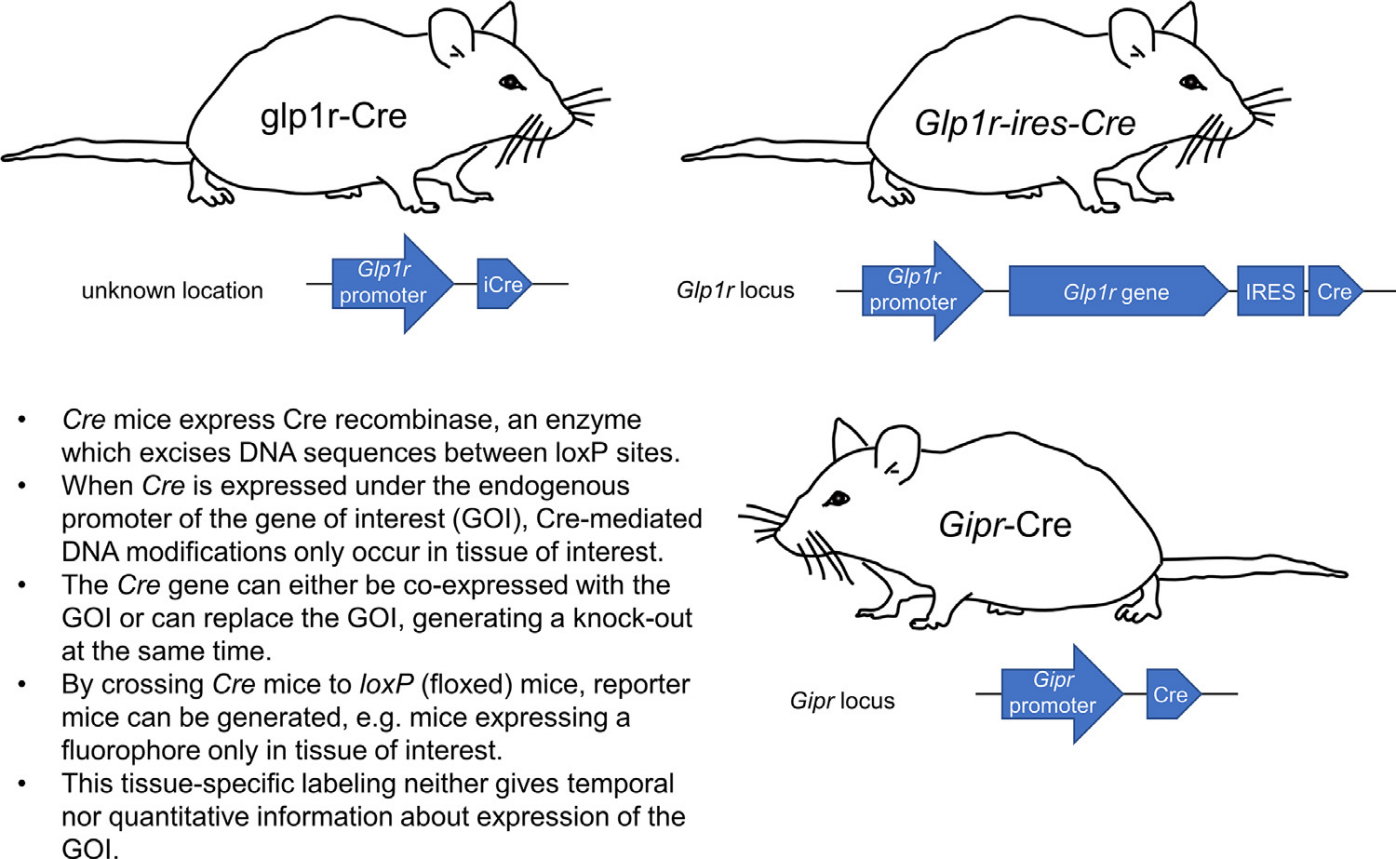
GLP1R and GIPR reporter mouse models. *Glp1rCre and Gipr-Cre* mouse models allow conditional labeling of *Glp1r*- and *Gipr*-expressing cells following Cre-dependent recombination of a fluorescent reporter allele. The different characteristics of the various models are detailed.

GIPR: *Gipr-Cre* mice have been recently reported in which the *Gipr* coding sequence was replaced by iCre in a BAC, following CRISPR-Cas9-mediated homologous recombination in one-stage fertilized embryos using sgRNAs targeting the wild-type *Gipr* gene [32]. Analysis of these animals showed that animals with a single *Cre* allele (i.e. heterozygous, missing a single *Gipr* allele) displayed normal body weight gain and fat mass [32]. Moreover, EYFP reporter expression showed overlap between *Cre* expression and known *Gipr*-expressing sites based upon mRNA expression (e.g. pancreatic alpha and beta cells, adipose tissue) [32]. While the animals are currently less well-validated than *Glp1rCre* animals, it can be assumed that some of the same advantages (e.g. fidelity, lineage tracing) and disadvantages (e.g. lack of information on protein levels) apply. An additional advantage of *Gipr-Cre* mice is that they can be bred as homozygotes to produce KO controls. The different *Glp1rCre* and *Gipr-Cre* mouse models are summarized in Fig. 1.

For all of the reporter mouse models mentioned, there is a need to more fully characterize Cre-recombination efficiency as well as expression patterns across the various tissues. This is particularly the case in the brain, where robust counter-staining methods (antibody, probe) work less effectively and RNAscope or similar approaches are needed.

## 6 Fluorescent agonist/antagonist probes

Current GLP1R antibodies have variable specificity and, except for the monoclonal antagonistic antibody, can only be used in fixed tissue. On the other hand, Cre reporter approaches allow labeling of *Gipr*- and *Glp1r*-expressing cells in live and fixed tissue, but do not report expression of the protein itself. Neither approach is able to provide information regarding how ligands access and then bind to GIPR/GLPR, which is important from a therapeutic standpoint. Another alternative for visualization of GLP1R/GIPR is thus the use of chemical probes, usually based upon orthosteric ligands furnished with a fluorophore for light-microscopy.

GLP1R: In their early iterations, fluorophore probes were generated by modifying the potent agonist Exendin4 (Ex4) at position K12 with VT680-NHS-ester, a near infrared fluorophore [57]. The probe, termed E4_K12_-Fl, was able to label GLP1R_HEK293 cells, MIN6 beta cells and pancreatic islets in vivo, overlapping with an insulin reporter or insulin protein [57]. The same authors used click chemistry on a K12-substituted Ex4 to install an azide-functionalized VT750 or Cy7 fluorophores, showing utility for estimating in vivo beta cell mass in NOD type 1 diabetes and insulinoma xenograft models [58, 59]. Demonstrating the importance of modification site, an Exendin4 (Ex4)-like neopeptide was substituted (or added) at positions 12, 27 and 40, before reaction with various NHS-fluorophores spanning green through near-infrared wavelengths [60, 61]. These studies showed that substitution/addition at position 12 and 40 were well-tolerated, that potency of the agonist depended on the fluorophore used as well as presence/absence of a *C*-terminal lysine, and that fluorescent probes can be used to purify pancreatic beta cells for sequencing [60, 61]. Subsequent studies reacted exendin4-Cys-amide with maleimide-Cy3 to generate Ex4-Cy3 [51]. This probe was able to label wild-type islets, co-localized with beta cells, but not alpha and delta cells, and a Cy5 version was found to be useful for whole pancreas optoacoustic imaging [51]. Notably, the authors showed that Ex4-Cy3-labeling was completely absent in *Glp1r*^−/−^ islets, demonstrating target specificity [51]. Ex4-FITC, Ex4-TMR (both K12-substituted) and lixisenatide‐FITC probes have also been described and were tested using pharmacology and SNAP_GLP1R binding 62, 63, 64. Lastly, fluorescent congeners have recently been produced for other GLP1R agonists, including liraglutide^594^, liraglutide^750^ and semaglutide^VT750^ [65, 66], all validated in *Glp1r*^−/−^ tissue.

The major advantage of such probes is that they allow one-step intense staining of live islets, free from background introduced by chemical fixation methods. An inherent disadvantage of these probes, however, is that fluorescent agonist probes by their nature strongly bind, activate and internalize GLP1R, and this can confound some experiments (e.g. those enriching GLP1R+ cells for transcriptomic analysis). To circumvent this issue and to more widely open up super-resolution imaging of endogenous GLP1R, we recently produced the LUXendins, which are based on the potent GLP1R antagonist Exendin9 (Ex9) [29]. By substituting the *C*-terminal (position 39) serine for a cysteine, a range of maleimide-fluorophores can be installed without any appreciable loss of potency versus native antagonist [29]. LUXendins endowed with tetramethylrhodamines (TMR), blinking cyanine5 (Cy5) and fluorogenic silicon rhodamine (SiR) allow a range of experiments, including widefield, confocal, intravital and stimulated emission depletion (STED) microscopy of GLP1R applied to both live and fixed islets (vide infra). Notably, LUXendins stain GLP1R at the cell surface, leading to clean membrane-bound signal. Using a novel CRISPR-deleted *Glp1r^−/−^* mouse line, as well as validated antibodies, LUXendins were shown to be highly specific for GLP1R [29]. No signal could be detected in *Glp1r^−/-^* islets suggesting that LUXendins do not promiscuously bind the glucagon receptor or GIPR [29]. The LUXendin family has now been expanded to encompass 7 different colors, spanning green to near-infrared wavelengths (CF488A, Cy3, TMR, CPY, Cy5, SiR and Cy7) [22]. A related antagonist probe also exists, exendin(9-39)Alexa Fluor 594, which has been validated in *Glp1r*^*−/−*^ tissue and which also displays clean membrane labeling when viewed at high, but not low, resolutions [66]. Exendin(9-39)-FITC has also been described, which labels HEK293-SNAP-GLP1R cells and out-competes unmodified Ex4 in equilibrium binding assays [67, 68].

It is worth noting that simply bolting fluorophores onto agonist or antagonist does not guarantee success. Different fluorophores possess different properties, which can influence pharmacology. Also, fluorophore properties can change in the tissue environment such that the best performing dye on paper or in vitro does not necessarily lead to the best or most specific signal. Thus, for any new compound, chemical characterization should be reported (HRMS), purity checked (HPLC), structures disclosed, and pharmacology and target specificity determined. Nonetheless, fluorescent GLP1R agonists and antagonists provide non-genetic, useful and specific tools for GLP1R visualization alongside reporter mice and antibodies.

GIPR: development of GIPR fluorescent probes is still a work in progress, hampered by the historical lack of potent and stabilized GIPR agonists that can be used in vitro. Moreover, GIPR peptide antagonists demonstrate poor *IC_50_*s for cAMP inhibition and are largely unsuitable for installation of fluorophores. However, Aib2-stablized GIP analogs represent strong candidates for fluorophore installation [16, 69], as well as recently reported short (GIP-532) and long (GIP-085) acting GIPR agonists [46], although any compound should be carefully validated using *Gipr-Cre* reporter and *Gipr^−/−^* mice.

**Fig. 2:**
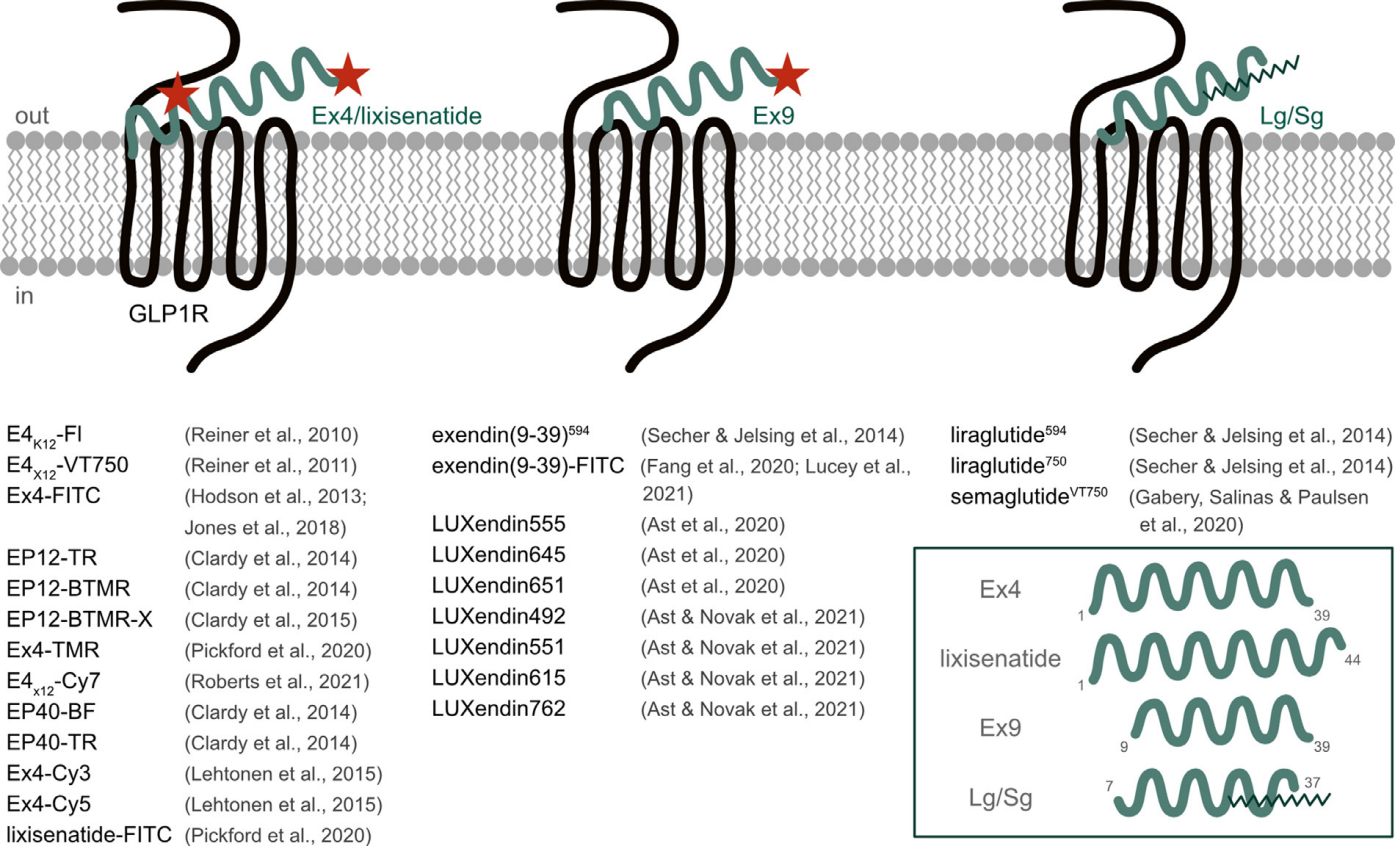
Agonist and antagonist chemical probes for GLP1R detection. Chemical probes based upon the agonists Exendin4(1-39) (Ex4)/lixisenatide/liraglutide (Lg)/semaglutide (Sg), or the antagonist Exendin9 (Exendin4(9-39); Ex9), can be used to visualize GLP1R in live and fixed tissue. The different fluorophore labeling strategies are shown and known chemical probes listed. Fluorophore position is not shown where compound characterization is not fully reported.

GLP1R agonist and antagonist probes are summarized in Fig. 2.

## 7 Fluorescent tags

**Fig. 3:**
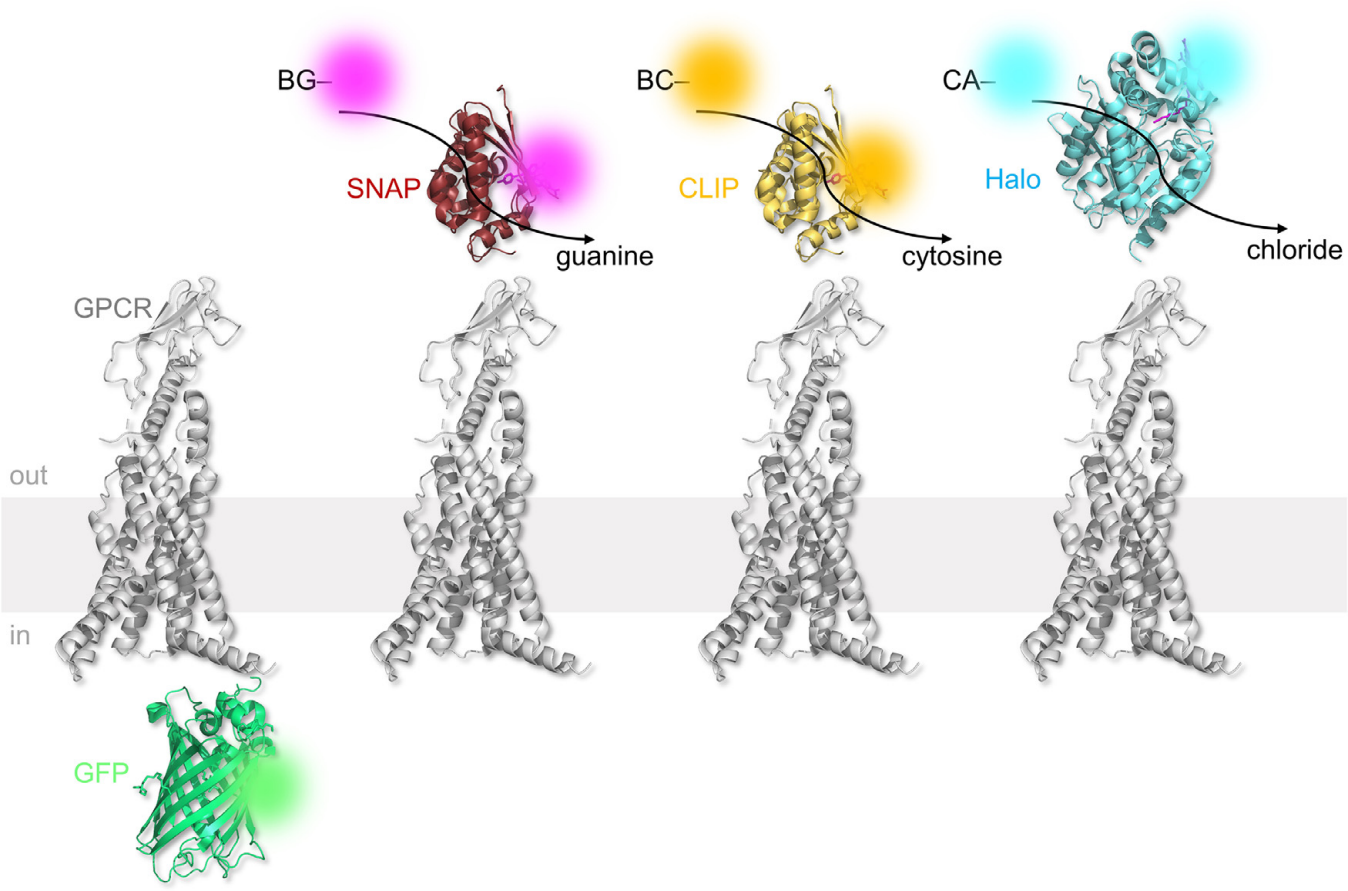
Fluorescent tagging and enzyme self-labeling strategies for GLP1R and GIPR. Fluorescent proteins (e.g. GFP) may be fused to the receptor of interest. Fluorescent proteins are bright and some can be photoconverted, which is useful for PALM microscopy. However, this approach is inherently less flexible than enzyme self-labeling. SNAP-, CLIP- and Halo-tags react with *O*^6^-benzylguanine- (BG-), *O*^2^-benzylcytosine- (BC-) and chloroalkane- (CA) linked substrates, respectively. Binding is covalent and a range of substrates can be flexibly attached to the receptor of interest, including fluorophores, metal ions or biotins. Advantageously, different receptor pools can be studied. pdb: 7lck (GLP1R); 5n9o (GFP); 3kzz (SNAP and CLIP); 6u32 (Halo).

Recombinant genetics has allowed fluorescent proteins to be incorporated into proteins, allowing their visualization using light microscopy [70]. A number of mutant GLP1R and GIPR constructs have been reported, which express GFP on the *C*-terminal end, thus allowing the receptor to be tracked during imaging experiments (Fig. 3). GLP1R_GFP and GIPR_GFP constructs have been used to understand trafficking and internalization in heterologous cell systems (e.g. HEK293 and adipocytes) 71, 72, 73, 74. While GLP1R_GFP/GIPR_GFP fusion proteins are reported to traffic to and from the membrane normally, efforts should be made to titrate the plasmid and phenotype the cell system, since high levels of GFP have been shown to lead to NADH-dependent superoxide (O_2_^•−^) and peroxide generation, as well as HIF1α stabilization [75]. Moreover, comparisons should be made with wild-type (but immunostained) and SNAP-tagged receptors in case of effects of fluorophore on trafficking dynamics. Another issue with GFP is that, by modern standards, it has poor quantum yield and brightness. However, newer, brighter and smaller fluorescent proteins exist (e.g. mNeon Green—3x brighter than GFP), which should allow improved GLP1R/GIPR detection. While their use is declining in favor of enzyme self-labels, fluorescently-tagged GLP1R/GIPR constructs are a mainstay of photoactivated localization microscopy (PALM), a super-resolution imaging modality which relies on photoconversion of fluorescent proteins (e.g. mEos4) or “caged” secondary antibody (e.g. anti-FLAG CAGE 500 [76]) between two states.

**Latest innovation for GLP1R/GIPR detection**

## 8 Enzyme self-labels

For most imaging applications, fluorescently-tagged constructs have been largely superseded by enzyme self-labels. The basic principle of enzyme self-labels is that they recognize and bind to a specific substrate, forming an irreversible covalent bond. So far, three major players have emerged: SNAP-tag, HaloTag and CLIP-tag. The SNAP-tag is an evolved *O*^6^-alkylguanine-DNA alkyltransferase mutant, which reacts with its substrate *O*^6^-benzylguanine, forming a covalent bond and liberating guanine [77]. CLIP-tag is an engineered version of SNAP-tag, which instead reacts with *O*^2^-benzylcytosine [78]. By contrast, the HaloTag is a haloalkane dehalogenase, which reacts with a chloroalkane to form a covalent alkyl-HaloTag product [79]. The three enzyme self-labels are largely orthogonal and can be used in the same experiment to identify multiple proteins (Fig. 3) [80].

Enzyme self-labels have a number of advantages as genetically-encoded tools versus fluorescent proteins: 1) they are small (SNAP and CLIP: ∼20 kDa; Halo: ∼30 kDa; GFP: ∼27 kDa) and interfere minimally with protein function; 2) they are highly flexible, permitting protein visualization with different fluorophores; 3) labeling is irreversible; 4) multicolor pulse-chasing experiments can be performed (e.g. to visualize old versus new receptor populations); and 5) experiments can be performed by labeling with versatile chemical compounds (e.g. biotin or tethered drugs [81, 82]). For these and other reasons, enzyme self-labels, in particular SNAP-tag and HaloTag, have gained traction for the study of GPCR signaling and trafficking. SNAP_GLP1R and SNAP_GIPR both exist and are well-characterized in heterologous cell systems [76, 81, 83, 84]. A number of complementary fluorescent SNAP-tag and HaloTag labels exist, spanning most colors in the visible range, as well as different imaging modalities. Most recently, we have developed a number of SNAP-tag and HaloTag fluorescent labels, which allow surface GLP1R populations to be selectively visualized using conventionally cell permeable dyes [85, 86]. Before this, surface labeling depended on the chemical properties of the dye itself, limiting the number of colors that could be used [86].

To date, enzyme self-labels remain largely restricted to cell lines where SNAP-, Halo- and CLIP-tagged GLP1R and GIPR are widely used, particularly for pharmacological assays (kinetics, coupling and potency) or single molecule imaging (dynamics and internalization). While SNAP-tags have been used to conditionally label cells, this was achieved using Rosa26SNAPCaaX reporter mice rather than labeling the endogenous protein itself [87]. However, CRISPR-Cas9 knock-in approaches open up the possibility to SNAP/CLIP/Halo-tag the endogenous *Glp1r*/*Gipr* loci in zebrafish, mouse and rat, and we expect these models to become available in the near future. The major advantage of using enzyme self-labels is that surface or total receptor pools can be visualized in live and fixed tissue at a chosen timepoint without interfering with orthosteric or allosteric binding modes. However, one has to keep in mind that an exogenous chemical (e.g. fluorophore) needs to be delivered.

**Super-resolution imaging of GLP1R/GIPR**

Due to their abundance and size, GPCRs are ideal candidates for detection using nanoscopic or super-resolution imaging. Three principal types of super-resolution microscopy exist, which are able to visualize fluorophores in the 40-70 nm range: stochastic optical reconstruction microscopy (STORM), PALM and STED microscopy. STORM and PALM depend upon stochastic single-molecule localization to surpass the diffraction limit of light, whereas STED relies on excitation and parallel de-excitation of fluorophores just outside of the focal spot with a donut beam. Each method is highly dependent upon specific fluorophores, which not only display high quantum yields, sufficient Stokes shifts and robustness towards bleaching, but come with unique properties. Thus, PALM requires photoconvertible proteins or caged antibodies [76], STORM needs blinking fluorophores, while STED depends upon robust dyes with good depletion performance.

**Fig. 4:**
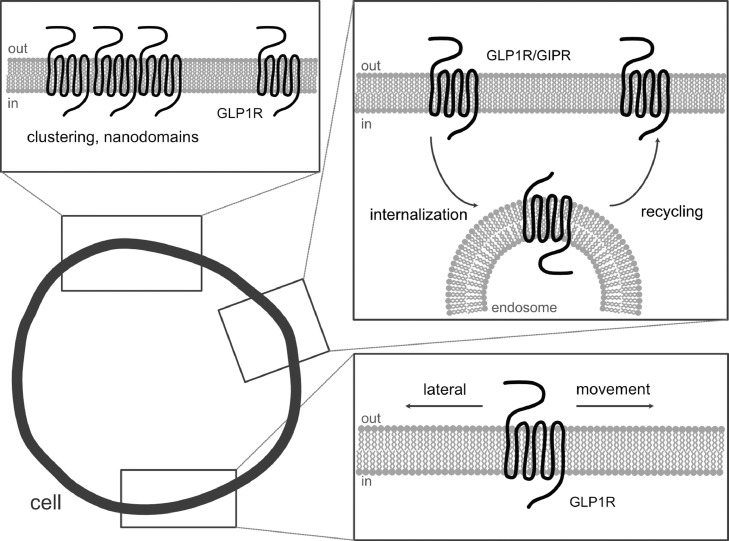
Super-resolution visualization of GLP1R/GIPR reveals new facets of their organization**.** By visualizing GLP1R/GIPR with validated super-resolution compatible reagents, higher organization can be appreciated, including clustering into membrane nanodomains, dynamic internalization and trafficking, and diffusion at the membrane (in their non-stimulated and stimulated states).

While super-resolution understanding of GLP1R/GIPR is still in its infancy, early studies have already provided insight into the higher-order organization of the receptors. Initial PALM-total internal reflection fluorescence (TIRF) studies of the GLP1R revealed ligand-induced clustering at the membrane [76]. Subsequent studies used STED to look at endogenous GLP1R, revealing the existence of membrane nanodomains in the unstimulated state [29]. Single molecule-localization microscopy showed that, even in their non-stimulated state, GLP1R are mobile at the membrane and can be classed according their diffusion rate [29, 76]. In the future, it will be interesting to understand more about how GLP1R higher organization influences ligand stimulation and signaling, and how this might change during disease as well as within the tissue context. Moreover, SNAP-GIPR and fluorescent GIPR agonists should allow the first super-resolution snapshots of GIPR. GLP1R/GIPR higher organization is summarized in Fig. 4.

## 9 Electron Microscopy of GLP1R/GIPR

While super-resolution fluorescence microscopy allows high-definition snapshots of fluorophores beyond Abbe's diffraction limit, electron microscopy is capable of magnifying ultrafine structures down to the single-digit nanometer level. Such approaches are only applicable in fixed cells, which are heavily processed before imaging and need to be sliced into sub-micron sections. To obtain contrast, heavy atoms are required as a stain, and are usually in the form of specific antibody-nanoparticle conjugates. Given the lack of specific antibodies, this is challenging and is further complicated by the antibody itself, which at ∼15 nm can interfere with nanometer scale structural measurements. To get around this obstacle, investigators have employed BG-SS-PEG4-biotin labeled SNAP_GLP1R constructs, which can be visualized following addition of gold-streptavidin. Such studies showed that distances between the gold particles, and hence SNAP_GLP1R, decreases with ligand-stimulation [76]. In a more recent iteration of this method, a genetically encoded tag has been described that allows the direct synthesis of gold nanoparticles by chemical treatment for electron microscopy [88]. Another method involves cloning an APEX2 tag, a peroxidase, onto the protein of interest, which generates an electron-dense osmiophilic polymer following application of diaminobenzidine and peroxide [89, 90]. Further tools are warranted to be able to use the power that contemporary techniques, including correlative light and electron microscopy (CLEM) and focused ion beam scanning electron microscopy (FIB-SEM), offer. First stabs into the direction of beta cell structures have been made with CLEM to visualize insulin granules with dual colors [91], the reconstruction of complete beta cells to highlight microtubule-organelle interactions and insulin granule distributions by FIB-SEM [92], as well as soft x-ray tomography [93]. Going forwards, cryo-CLEM and cryo-FIB-SEM should allow imaging of GLP1R/GIPR in samples without the need for dehydration (which can introduce artefacts) and resin-embedding (not suitable for some samples) [94, 95]. Due to the resolutions achieved, electron microscopy allows quantification of GLP1R/GIPR numbers, as well as their localization within the cell (membrane, endosome, cytosol).

## 10 Outstanding questions

Detecting and visualizing GLP1R and GIPR remains a challenge, as for most GPCRs. Despite the translational importance of GLP1 and GIP biology [15, 96], the development of specific reagents for GLP1R/GIPR detection has not kept pace with drug development. Without being able to accurately detect GLP1R/GIPR in time and space, we are missing critical information pertaining to the impact of their signaling on cell function. It should be clear that an abundance of caution is required when using new reagents, particularly antibodies from commercial sources. Any new reagent should be extensively validated and characterized, and the models to do this now exist, including GLP1R/GIPR knockout animals and beta cell lines. Alongside conventional reagents, a number of new innovations are available/under development, including reporter animals, SNAP/Halo-tagged receptors and novel fluorescent probes. Moreover, recent developments in imaging are beginning to provide unprecedented insight into the high-resolution biology of GLP1R/GIPR distribution and signaling. Nonetheless, a number of pertinent questions remain to be addressed. Firstly, what is the exact localization of GLP1R and particularly GIPR in target tissues? Secondly, if GLP1R and GIPR are not localized in a ‘target’ tissue such as the liver, how are they able to exert indirect influence over this tissue (e.g. via effects on immune cells, for example CD8+ and γδTCR located in the liver [23])? Thirdly, do different populations of GLP1R and GIPR signal similarly in different tissues? Fourthly, what is the relevance of the higher-order organization of GLP1R and GIPR and is this influenced differentially by the various agonists? We predict that, with the right tools and techniques in place, the GLP1/GIP field will be able to address such questions and in doing so define novel mechanisms of action, with translational impact across metabolic, inflammatory and neurological disease.

## 11 Guidance on best practice for detection of GLP1R and GIPR

Any new reagent should be treated as non-specific until properly validated. Reagent validation will depend upon cell type, tissue and species:

1) For studies in heterologous cell systems, the reagent should be tested in cells expressing SNAP-, Halo- or CLIP-GLP1R/GIPR, orthogonally labeled using fluorophore. No signal should be detected in cells without the SNAP/Halo/CLIP_GLP1R/GIPR construct. Agonist/Antagonists should show similar *EC*_*50*_/*IC*_*50*_ to unmodified ligand.
2) For rodent tissue, GLP1R/GIPR^−/−^ tissue or GLP1R/GIPR^−/−^ beta cell lines should be used, showing absence of staining/labeling. Antibodies can be further cross-validated using fluorescent agonist or antagonist probes with known specificity (and vice versa) and/or *Glp1rCre* reporter animals. While there is high homology between mouse and rat GLP1R/GIPR, reagents intended for experiments in rat should ideally be tested against overexpressed rat GLP1R/GIPR and/or rat INS1 832/3 GLP1R/GIPR^−/−^ cells.
3) For human tissue, reagents should be tested in cells transfected with mock or hGLP1R/hGIPR, with absence of staining in mock cells. Alternatively, signal should co-localize with labeled SNAP-, Halo- or CLIP-hGLP1R/hGIPR. Antibodies can be further cross-validated using fluorescent agonist or antagonist probes with known specificity (and vice versa). Further confidence is gained by purifying the ‘labeled’ cell type and amplifying GLP1R/GIPR using primers against the ORFs versus known positive controls (Brunner's gland, islets).

## 12 Search strategy and selection criteria

Information for the current review article was derived from the authors’ combined expertise and knowledge of the field, together with targeted interrogation of PubMed using GLP1R and GIPR as primary search terms. Other experts in the field were also consulted as part of our efforts to identify specific reagents for detecting GLP1R and GIPR.

## 13 Contributors

J.A., J.B. and D.J.H. contributed to the literature search, writing of the paper and design/production of figures. All authors read and approved the final version of the manuscript

## Declaration of competing interest

J.B. and D.J.H. have a licensing deal with Celtarys Research for production and supply of LUXendins and other fluorophore-conjugated peptidic pharmacophores.
